# Recruiting Adolescent and Young Adult Cancer Survivors for Patient-Reported Outcome Research: Experiences and Sample Characteristics of the SURVAYA Study

**DOI:** 10.3390/curroncol29080428

**Published:** 2022-07-29

**Authors:** Carla Vlooswijk, Lonneke V. van de Poll-Franse, Silvie H. M. Janssen, Esther Derksen, Milou J. P. Reuvers, Rhodé Bijlsma, Suzanne E. J. Kaal, Jan Martijn Kerst, Jacqueline M. Tromp, Monique E. M. M. Bos, Tom van der Hulle, Roy I. Lalisang, Janine Nuver, Mathilde C. M. Kouwenhoven, Winette T. A. van der Graaf, Olga Husson

**Affiliations:** 1 Research and Development, Netherlands Comprehensive Cancer Organisation, 3511 DT Utrecht, The Netherlands; c.vlooswijk@iknl.nl (C.V.); l.vandepoll@iknl.nl (L.V.v.d.P.-F.); e.derksen@iknl.nl (E.D.); 2 Department of Psychosocial Research and Epidemiology, Netherlands Cancer Institute, 1066 CX Amsterdam, The Netherlands; sh.janssen@nki.nl; 3 Department of Medical and Clinical Psychology, Tilburg University, 5037 AB Tilburg, The Netherlands; 4 Department of Medical Oncology, Netherlands Cancer Institute-Antoni van Leeuwenhoek, 1066 CX Amsterdam, The Netherlands; m.reuvers@nki.nl (M.J.P.R.); j.kerst@nki.nl (J.M.K.); w.vd.graaf@nki.nl (W.T.A.v.d.G.); 5 Department of Medical Oncology, University Medical Center, 3584 CX Utrecht, The Netherlands; r.m.bijlsma@umcutrecht.nl; 6 Department of Medical Oncology, Radboud University Medical Center, 6525 GA Nijmegen, The Netherlands; suzanne.kaal@radboudumc.nl; 7 Dutch AYA ‘Young and Cancer’ Care Network, Netherlands Comprehensive Cancer Organisation, 3511 DT Utrecht, The Netherlands; 8 Department of Medical Oncology, Amsterdam University Medical Centers, 1105 AZ Amsterdam, The Netherlands; j.m.tromp@amsterdamumc.nl; 9 Department of Medical Oncology, Erasmus MC Cancer Institute, Erasmus University Medical Center, 3015 GD Rotterdam, The Netherlands; m.bos@erasmusmc.nl; 10 Department of Medical Oncology, Leiden University Medical Center, 2333 ZA Leiden, The Netherlands; t.van_der_hulle@lumc.nl; 11 Department of Internal Medicine, GROW-School of Oncology and Reproduction, Maastricht UMC+ Comprehensive Cancer Center, 6229 HX Maastricht, The Netherlands; roy.lalisang@mumc.nl; 12 Department of Medical Oncology, University Medical Center Groningen, 9713 GZ Groningen, The Netherlands; j.nuver@umcg.nl; 13 Department of Neurology, Amsterdam UMC, Amsterdam University Medical Centers, Location VUmc, 1081 HV Amsterdam, The Netherlands; m.kouwenhoven@amsterdamumc.nl; 14 Department of Surgical Oncology, Erasmus MC Cancer Institute, Erasmus University Medical Center, 3015 GD Rotterdam, The Netherlands; 15 Division of Clinical Studies, Institute of Cancer Research and Royal Marsden NHS Foundation Trust, London SM2 5PT, UK

**Keywords:** adolescents and young adults with cancer (AYAs), population-based research, health-related outcomes, non-participation, recruitment strategies, patient-reported outcomes

## Abstract

Background: Participation of Adolescents and Young Adults with cancer (AYAs: 18–39 years at time of diagnosis) in patient-reported outcome studies is warranted given the limited knowledge of (long-term) physical and psychosocial health outcomes. We examined the representativeness of AYAs participating in the study, to observe the impact of various invitation methods on response rates and reasons for non-participation. Methods: A population-based, cross-sectional cohort study was performed among long-term (5–20 years) AYA cancer survivors. All participants were invited using various methods to fill in a questionnaire on their health outcomes, including enclosing a paper version of the questionnaire, and sending a reminder. Those who did not respond received a postcard in which they were asked to provide a reason for non-participation. Results: In total, 4.010 AYAs (response 36%) participated. Females, AYAs with a higher socio-economic status (SES), diagnosed more than 10 years ago, diagnosed with a central nervous system tumor, sarcoma, a lymphoid malignancy, stage III, or treated with systemic chemotherapy were more likely to participate. Including a paper questionnaire increased the response rate by 5% and sending a reminder by 13%. AYAs who did not participate were either not interested (47%) or did want to be reminded of their cancer (31%). Conclusions: Study participation was significantly lower among specific subgroups of AYA cancer survivors. Higher response rates were achieved when a paper questionnaire was included, and reminders were sent. To increase representativeness of future AYA study samples, recruitment strategies could focus on integrating patient-reported outcomes in clinical practice and involving AYA patients to promote participation in research.

## 1. Introduction

Adolescents and young adults (AYAs) are recognized as a distinct population within the oncology community due to the unique challenges they encounter, including delayed diagnosis, lack of progress in treatment, and psychosocial issues [1,2,3,4,5]. The US National Cancer Institute proposed defining AYAs as those aged 15–39 years at initial diagnosis, but also concluded that this age range should be flexibly applied, depending on specific research questions and the health care delivery system [4]. In the Netherlands, care for cancer patients is categorized into centralized pediatric oncology for children (0–18 years) and medical oncology for adults (≥18 years). Therefore, in the Netherlands, AYAs are defined as those aged 18 to 39 years at initial cancer diagnosis and can make use of the age-specific care provided in AYA expert centers nationally coordinated by the Dutch AYA health care network. Although cancer is a disease predominantly affecting older adults, on average 3500 AYAs were diagnosed with cancer in the Netherlands annually in the period between 2010 and 2016 [6]. Survival has been increasing in the AYA population. With a 5-year relative survival of >80%, most AYAs have a long life ahead of them. 

In past decades, more and more attention has been paid to the unique clinical needs of AYA cancer patients and, in parallel, the development of specialized AYA guidelines and cancer centers internationally [7,8,9,10,11]. The unique needs of AYAs with cancer include dealing with issues such as fertility, social isolation, family functioning, employment, and financial toxicity [10,11,12]. Studies that address long-term health issues show that AYA cancer survivors are at greater risk for late effects, such as cardiomyopathy, hearing loss, stroke, thyroid disorders, and diabetes than the general population [13]. Additionally, regarding psychological aspects, AYA cancer survivors are at greater risk of worse mental health than their counterparts, even more than 6 years after completion of treatment [14]. 

To increase knowledge about the long-term health issues among AYAs, it is important to perform studies among AYAs. However, research has shown that AYA cancer patients have participated less often in clinical trials than younger and older patients [15]. Although the reasons for low clinical trial enrollment among AYAs are not well understood, it will likely be a combination of treatment setting and provider factors (community settings with limited access to trials; knowledge of available trials), with patient-(concerns, knowledge, attitudes, personal conflicts, and socioeconomic factors including underinsurance) and system-level factors (age restrictions; trial availability) [15,16,17]. Clinical trial participation of AYAs is very important, because currently there is limited knowledge about the effectiveness of treatments for AYA, which has been described as one of the reasons for the limited progress in survival in this age group [18].

Next to clinical trials, patient-reported outcome (PRO) studies can provide relevant information on health outcomes; however, participation of AYAs in PRO studies is often low, with response rates ranging from 25% to 52% [19,20,21,22,23,24].

In these previously conducted PRO studies, response rates improved by using personal invitation methods and patients preferred paper-pencil rather than online questionnaires [19]. These studies also showed that certain AYA subgroups were less likely to participate. For instance, males and Hispanics less often participate in PRO research than females and non-Hispanic whites [25,26]. In addition, AYAs diagnosed with a melanoma or gynecologic cancer were slightly underrepresented. Reasons for non-participation in observational PRO studies among AYA were not studied before and therefore remain largely unknown.

The burden of adverse long-term health outcomes of cancer and its treatment in AYA cancer survivors highlighted the importance to get more insight into AYA patient subgroups that are more susceptible to specific poor long-term health issues [10]. Therefore, we conducted an observational population-based, cross-sectional cohort study among 5–20-year survivors of AYA cancer; the SURVAYA study (health-related quality of life and late effects among SURVivors of cancer in Adolescence and Young Adulthood). Most research focusing on response rates has been done among AYA in treatment or shortly after. The SURVAYA study provides an optimal opportunity to examine the best way to approach long-term AYA cancer survivors for PRO research.

The secondary aims of the SURVAYA study were to (1) examine representativeness of the study sample regarding sociodemographic and clinical characteristics; (2) examine the impact of different invitation methods on response rate; and (3) describe reasons for non-participation. 

## 2. Materials and Methods

### 2.1. Setting and Population

An observational population-based, cross-sectional cohort study was performed among AYA patients (18–39 years old at time of cancer diagnosis) registered within the Netherlands Cancer Registry (NCR). The NCR is a population-based registry that covers the total Dutch population of more than 17 million people. Patients diagnosed with cancer between 2000 and 2015 (except stated otherwise between brackets) and treated in the Netherlands Cancer Institute (1999–2014) or one of the university medical centers (University Medical Center Utrecht (1999–2014), Academic Medical Center (1999–2014), Erasmus Medical Center, Maastricht University Medical Center, Radboud University Medical Center, VU University Medical Center, Leiden University Medical Center, and University Medical Center Groningen (1999–2015) were included. 

Patients with clinically diagnosed cancer (without histological diagnosis) were excluded. In addition, the following diagnoses were excluded for multifactorial reasons, mainly, based on very good prognosis or extreme rarity of the tumor in this age group (and therefore it is sometimes unclear to the patient that he/she has a form of cancer): neuroendocrine tumors of the gastrointestinal tract, unknown primary site, skin adnexal carcinoma, unspecified skin carcinoma, squamous cell carcinoma of skin, basal cell carcinoma, dermatofibrosarcoma, Kaposi sarcoma, atypical lipoma, atypical chondroma, placental trophoblast tumors, cutaneous lymphomas, and unknown tumor types. The SURVAYA study was approved by the Netherlands Cancer Institute Institutional Review Board (IRB-IRBd18122) and registered within clinical trial registration (NCT05379387).

### 2.2. Data Collection

Data collection was conducted between May 2019 and June 2021 within PROFILES (Patient Reported Outcomes Following Initial treatment and Long-term Evaluation of Survivorship) [27]. PROFILES is a registry to study the physical and psychosocial impact of cancer and its treatment from a dynamic, growing population-based cohort of both short- and long-term cancer survivors. A linkage with the Dutch municipal records database was established to obtain up-to-date addresses and to know patients are alive at the moment of inviting.

All participants were informed about the study via a letter from their (ex-) attending medical specialist. Three various ways of invitation methods were used and categorized into the following groups: the paper-optional group, no reminder group, and paper-included group.

#### 2.2.1. Paper-Optional Group (*N* = 8291)

The study invitation consisted of a letter with a secure link to a web-based informed consent form, online questionnaire, and log-in instructions. A reply card with a pre-stamped return envelope was also included, to give participants the option to request a paper version of the questionnaire. A reminder was sent to the paper-optional group.

#### 2.2.2. No Reminder Group (*N* = 1671)

The study invitation was the same as for the paper-optional group; however, no reminder was sent to assess the impact of a reminder.

#### 2.2.3. Paper Included Group (*N* = 1334)

This group received the same invitation letter with a secure link to a web-based informed consent, online questionnaire and log-in instructions, but with a paper questionnaire and a pre-stamped return envelope also included. A reminder was sent. 

The reminder for the paper-optional group and paper-included group was anticipated to be sent within 3 months after the first invitation; however, due to COVID-19, it was not possible to send invitations in the hospitals and therefore the reminder was sent within a timeframe of 2–7 months. The reminder letter consisted of a link to a web-based informed consent form and online questionnaire, and a postcard which could be used to indicate their reason(s) if patients did not want to participate in the study. 

### 2.3. Measures

Sociodemographic and clinical characteristics were available from the NCR. 

Sociodemographic data included gender, age and social-economic status (SES). SES scores arise from the standardized income per household, extracted from four numbers and letters of the Dutch postal code of the Netherlands Institute for Social Research [20]. The scores were decoded into deciles, which were consequently classified as low (deciles 1,2,3), medium (deciles 4,5,6), and high (deciles 7,8,9) SES. 

Clinical data included tumor type, tumor stage, primary treatments received, and time since diagnosis. Tumor type was classified according to the third International Classification of Diseases for Oncology (ICDO-3) [28]. Cancer stage was classified according to TNM or Ann Arbor Code (Hodgkin lymphoma and Non-Hodgkin lymphoma) [29]. TNM 5 was used for patients diagnosed from 1999 to 2002, TNM 6 for patients diagnosed from 2003 to 2009, and TNM 7 was used for patients diagnosed from 2010 to 2015. For tumors in the central nervous system, neuroblastomas, paraganglioma, extragonadal / extracerebral germ cell tumors, plasma cell tumors, myeloid hematological malignancies such as acute and chronic myeloid leukemia, myeloproliferative neoplasms, and myelodysplastic syndrome, tumor stage was not determined nor registered.

Information on marital status and educational level were patient-reported via the questionnaire (data only available for respondents). 

Reasons for not wanting to participate in the study were patient-reported. AYAs could give multiple answers via the following given response categories (determined a priori with input from AYAs with cancer): I am not interested in the research, I don’t want to think about cancer, I prefer an in person invitation, I have never considered myself as an AYA young adult cancer patient, I don’t see the added value of this research, I see no incentive or benefit of participating in this research, the questionnaire is too long, I have participated in research too many times, I am too busy, or an open-answer option.

### 2.4. Statistical Analyses

Statistical analyses were conducted using SAS version 9.4. (SAS Institute, Cary, NC, USA). All differences with a *p*-value < 0.05 were considered statistically significant. For the baseline characteristics, frequencies with percentages and means with standard deviations were used to describe the variables, and Chi-square tests and independent t-tests were used to test the differences between participants and non-respondents. In a multivariable logistic regression, associations between socio-demographic (age, gender, SES) and tumor characteristics (cancer type, stage, primary treatments received and time since diagnosis), and response were determined. Frequencies with percentages were used to describe the self-reported reasons of AYA non-responders and response rate of the paper-optional group, paper-included group, and no reminder group.

## 3. Results

**Figure 1:**
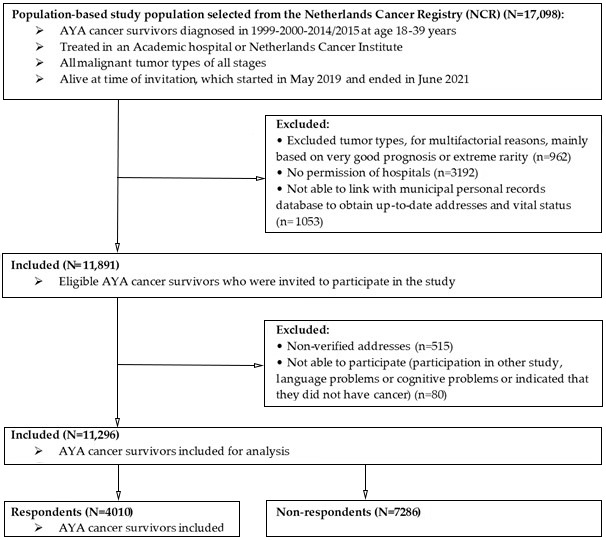
Flowchart of study participants in the SURVAYA study.

In total, 17,098 AYA cancer patients were identified via the Netherlands Cancer Registry, whereof 11,296 were invited to participate in the study (Figure 1). Reasons to exclude patients (*n* = 5802) were not having permission from the hospital to invite patients for the study (55%), or not being able to obtain up-to-date vital status and addresses because it was not possible or not allowed to link with municipal personal records database (18%). A total of 4,010 AYAs completed the questionnaire, which resulted in an overall response rate of 36%.

Table 1 describes the characteristics of the total AYA cancer population. Of the total population-based study population, 59% were females, average age of diagnosis was 31.5 years, and time since diagnosis was 12.2 years. Breast cancer (20%), germ cell tumors (16%) and lymphoid hematological malignancies (14%) were the most common tumor types. The supplementary materials (Table S1) shows the different tumor types divided into smaller subgroups.

### 3.1. Representativeness Study Sample

In multivariable logistic regression analysis, females were more likely to participate than males (Table 2). AYAs who were diagnosed more than 10 years ago were more likely to participate compared with AYAs who were diagnosed 5–10 years ago. AYAs with an intermediate or high SES were more likely to participate, compared with AYAs with a low SES. Moreover, compared with AYAs with breast cancer, AYAs diagnosed with cancer in the central nervous system, bone and soft tissues, or with a lymphoid hematological malignancy were more likely to participate. AYAs diagnosed with stage III cancer were more likely to participate than AYAs diagnosed with stage I cancer. AYAs treated with chemotherapy were also more likely to participate compared with AYAs who did not receive chemotherapy.

### 3.2. Methods of Invitation

**Figure 2:**
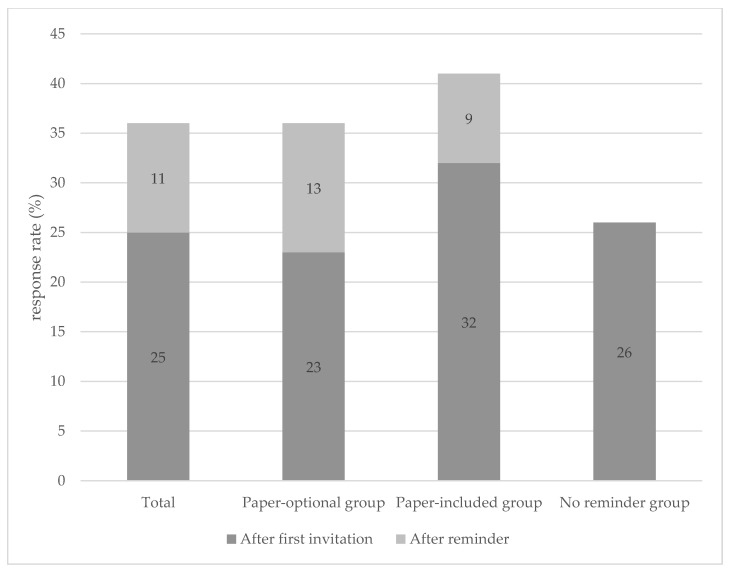
Response rates of the paper-optional group, paper-included group, and no reminder group.

The overall response rate was 36%. To be more specific, 25% of the AYAs responded to the initial invitation, 11% after a reminder was sent (Figure 2). Of the total invited AYA cancer patients in the paper-optional group, 3041 (36%) responded, whereof the reminder still yielded 13% of the response rate. The highest response rate (*n* = 544, 41%) was achieved in the paper-included group (32% after initial invitation and the remaining 9% after the reminder). The lowest response rate (*n* = 429, 26%) was shown in the group to which no reminder was sent. 

### 3.3. Reasons of Non-Participation

Of the non-respondents, 765 (11%) AYAs returned the postcard upon which they indicated (multiple) reason(s) for why they did not participate in the SURVAYA study. Nearly half of the AYAs (49%) who completed the postcard were not interested in participating in the study (Table 3). Furthermore, AYAs did not want to participate because they did not want to think about cancer (32%), were too busy (19%), considered the questionnaire as too long, personal or difficult (13%), and/or had never considered themselves as a young adult cancer patient (13%). The open answer category revealed additional reasons, such as privacy aspects, study participation invitation mail was not appreciated by AYAs, or AYAs were not capable to participate due to sickness or language problems.

## 4. Discussion

This large observational population-based, cross-sectional cohort study, addressing (sub)groups that are less inclined to participate, possible reasons for this and different ways of recruiting patients, recruited more than 4000 long-term AYA cancer survivors. Males and patients with a lower SES, diagnosed less than 10 years ago, having a lower disease stage, and not treated with chemotherapy were less likely to participate, as well as patients with specific tumor types. Paper questionnaires and reminders both had a positive impact on response rates. Reasons for non-participating in the study mainly included not being interested in the study or not wanting to be reminded of cancer.

In terms of the characteristics and representativeness of the SURVAYA study sample, similar to our findings, in previous studies among AYAs, males were less likely to participate in research [14,19,25,30]. In general, in health (behaviour) research, males are less likely to participate [31]. Reasons for lower participation among males are that they were generally less interested in participating than females, and that they were more likely to decline research participation due to time constraints [32]. Ryan et al. found that response rate among males improved substantially through the use of targeted Facebook advertising, incorporating features such as using images of men that appealed to leadership themes and using concise text [33]. In addition, male enrolments increased by asking female participants to invite males. 

AYAs who were diagnosed with cancer more than 10 years ago were more likely to participate. AYAs who are more than 10 years diagnosed with cancer are more often older adults. We hypothesize that their willingness to take part could have to do with life stage. It is probably harder to be closer to diagnosis and treatment and juggling with competing demands. 

Contrary to our findings, AYAs diagnosed with melanoma were underrepresented in previous research [14,25]. AYAs diagnosed with cancer in the central nervous system, bone and soft tissues, and with a lymphoid hematological malignancy were more likely to participate compared with AYAs with breast cancer. A possible explanation for this difference could be that patients diagnosed with less common cancer types probably are less often invited for participation in research. In addition, they might experience more consequences from their cancer diagnosis and treatment and might be more willing to tell their story, which could result in higher response rates among these patients. 

Patients with a low SES have shown to be largely underrepresented in research [34]. Barriers for participation of low SES patients could be language barriers, low health literacy, and distrust of the healthcare system [35]. Adults with a low health literacy were less interested in participating in research, probably because of the difficulties associated with understanding and skills needed to complete questionnaires [36]. Unfortunately, we did not have data on ethnicity, as these data are not collected by the NCR or in electronic patient files. We know from literature that Hispanic black and Hispanic patients were less likely to participate than non-Hispanic white patients as well [19,26].

In contrast to what was found in an older cancer population, we found that AYAs who were treated with chemotherapy were more likely to participate in the study [34,37]. Possibly, because AYAs who received chemotherapy experience daily long-term health consequences from their cancer diagnosis and treatment and were therefore more willing to participate. 

Our observed participation rate is, on average, similar compared with studies among AYA populations using similar recruitment strategies [21,22,23,24]. Given the younger age of this study population compared with the general cancer population, it is expected that they experience less problems or barriers with completing questionnaires online. However, the response rate improved by including a paper version of the questionnaire. Researchers from the AYA HOPE study found the same and explained this by the fact that unless thrown away, a questionnaire on paper is a constant reminder, whereas a computer/telephone can be turned off [38]. Sending a reminder resulted in a higher response rate and this is consistent with previous studies [39,40,41]. Among adolescents, in a study of Richards et al., personal contact seems to be effective. The initial response rate of 20% was improved by adding follow-up mailing (31%) and after conducting the consent process and questionnaire by phone (61%) [39].

To the best of our knowledge, no previous studies have assessed self-reported reasons for non-participation in PRO research among AYAs. Studies among the general population identified absence of interest and time constraints as reasons for non-participation in research, which was also found in our study [42,43,44]. Although patients did not want to participate in our study, they took the effort and time to return the postcard with reason(s) for non-participating. Remarkably, in our study, 13% of the AYAs did not consider themselves as a young cancer patient. A possible explanation could be that a significant part of the participants outgrew the AYA age and mistakenly did not consider themselves as an AYA. When approaching AYAs for future studies regarding long-term outcomes, it is important that participants feel connected and can identify with the target population of the study. In recent decades, the oncology community became aware of the gap in the care needs and outcomes of AYA patients [45]. Globally, but also in the Netherlands, development of AYA programs has flourished. This evolution in the field of AYA oncology could increase awareness and knowledge among future AYAs about age-specific care and research, and could thereby increase their willingness to participate in AYA research.

This study was limited by the cross-sectional design, limiting the determination of attrition rates. Study participants were diagnosed with cancer 5-20 years ago, which might have led to feeling less connected and attracted to participate in this study. Differentiating reasons of non-participation by different SES groups was not possible since patients had not given permission for this. It also remains unknown what the impact of COVID-19 is on the SURVAYA study. We could imagine that COVID-19 has had an impact on some of the outcomes of the SURVAYA study. We will examine this in a separate manuscript. 

The NCR allowed us to identify more than 17,000 cases of cancer among AYA aged 18–39 years between 1998 and 2015 in the participating hospitals. The NCR did not only act as a sampling frame, but also made it possible to perform a non-responder analysis, which gave insight into certain subgroups who are less likely to participate in observational research. In a previous study among the general cancer population, older patients with poorer Health-related Quality of Life (HRQoL) were less likely to participate, whereas younger patients with poorer HRQoL were more likely to participate [27,30]. To obtain more representative data, future studies should focus on personalized recruitment strategies to reach those who are less likely to participate, such as males and AYAs with lower SES. By performing qualitative research via focus groups and interviews, we could gain more insight into attitude towards participation in research, preferences in research type and invitation methods in specific subgroups of AYAs.

AYAs themselves might play an important role to stimulate and encourage fellow AYAs to participate in research. AYAs are widely acknowledged as key stakeholders in oncology and being used by research committees and advisory boards [44]. Involvement of AYA patient experts could bridge the gap between patient and research(ers). AYA patient experts could align research as closely as possible with the target group and help enhance recruitment, especially those who are less likely to participate (e.g., males, AYAs with low SES, ethnic minorities) [44]. Another option to get more PRO data of AYAs is better alignment between research and clinical care. PROs are more and more integrated in clinical practice. Moreover, PROs are more and more integrated in clinical studies, which reported high participation rates [46]. Ideally, PROs are used and aligned between research, health care and policy initiative, and adapted based on purpose. It is important that age-specific themes (e.g., fertility, body image, sexual health, financial security, life plans (educational and employment goals), and independence) are identified and described in a core outcome set and implemented in care and research [47,48].

## 5. Conclusions

In summary, the SURVAYA study recruited a large sample of AYA cancer survivors, however several differences were found between respondents and non-respondents based on registry data. Future studies should put effort into recruitment strategies, such as involving AYA patient experts as research partners to reach and encourage AYAs who are less likely to participate, such as males and AYAs with a low SES.

## Figures and Tables

**Table 1:** Socio-demographic and tumor characteristics of the total population-based population, and divided in respondents, non-respondents, and excluded.

		Total Population-Based Population		Respondents		Non- Respondents		Excluded		*P*-Value (Non- Respondents vs. Respondents)
		*N* = 17,098		*N* = 4010		*N* = 7286		*N* = 5802		
		*n*	%	*n*	%	*n*	%	*n*	%	
Gender	Male	6997	41	1549	39	3082	42	2366	41	0.0001
	Female	10,101	59	2461	61	4204	58	3436	59	
Age (at diagnosis), (mean (sd))		31.5 (5.9)		31.6 (5.9)		31.4 (5.9)		31.5 (5.8)		
	18–24 years	2672	16	613	15	1168	16	891	15	0.1354
	25–34 years	7782	46	1786	45	3328	46	2668	46	
	35–39 years	6644	39	1611	40	2790	38	2243	39	
Time since diagnosis, (mean (sd))		12.2 (4.6)		12.4 (4.5)		11.6 (4.4)		12.8 (4.8)		
	5–10 years	6362	37	1386	35	2990	41	1986	34	0.0001
	≥11–15 years	558	33	1397	35	2496	34	1687	29	
	≥16–20 years	5156	30	1231	31	1806	25	2119	37	
Social economic status	Low	3161	19	544	14	1510	21	1107	19	0.0001
	Intermediate	5427	32	1236	31	2354	32	1837	32	
	High	8456	50	2220	56	3417	47	2819	49	
Type of cancer	Head and neck	570	3	124	3	305	4	141	2	0.0001
	Colon and rectal	368	2	82	2	151	2	135	2	
	Digestive tract, other	262	2	31	1	63	1	168	3	
	Respiratory tract	166	1	30	1	71	1	65	1	
	Melanoma	1221	7	290	7	617	8	314	5	
	Skin, other	105	1	0	0	0	0	105	2	
	Breast	3346	20	944	24	1553	21	849	15	
	Female genitalia	2073	12	445	11	878	12	750	13	
	Male genitalia	24	0	6	0	12	0	6	0	
	Urinary tract	190	1	46	1	105	1	39	1	
	Thyroid gland	1054	6	248	6	468	6	338	6	
	Central nervous system	545	3	150	4	231	3	164	3	
	Bone or soft tissue sarcoma	1256	7	172	4	291	4	793	14	
	Germ cell tumors	2743	16	692	17	1394	19	657	11	
	Lymphoid hematological malignancies	2339	14	591	15	903	12	845	15	
	Myeloid hematological malignancies	671	4	148	4	226	3	297	5	
	Other	165	1	11	0	18	0	136	2	
Tumor stage	I	7758	45	1726	43	3480	48	2552	44	0.0001
	II	388	23	1063	27	1850	25	967	17	
	III	1994	12	573	14	923	13	498	9	
	IV	618	4	179	4	298	4	141	2	
	Missing	2848	17	469	12	735	10	1644	28	
Primary treatment modality	Surgery	13,145	77	3126	78	5863	81	4156	72	0.0017
	Chemotherapy	8199	48	2239	56	3632	50	2328	40	0.0001
	Radiotherapy	7681	45	1902	47	3293	45	2486	43	0.0213
	Hormone therapy	1734	10	484	12	786	11	464	8	0.0382
	Targeted therapy	1119	7	308	8	514	7	297	5	0.2178
	Stem cell therapy	523	3	142	4	207	3	174	3	0.0392
Marital status (at time of questionnaire)		Partner	NA	NA	3333	83	NA	NA	NA	NA
Education level	No education or primary education	NA	NA	28	1	NA	NA	NA	NA	
	Secondary education			266	7					
	Secondary (vocational) education			1456	36					
	Higher (vocational) education			1374	34					
	University education			878	22					
Mode of completion	Paper	NA	NA	647	16	NA	NA	NA	NA	
	Online			3363	84					

**Table 2:** Odds ratios (OR) of respondents versus non-responders, multivariable logistic regression.

		Respondents		Non-Respondents		Odds of Respondents vs. Non-Respondents		
		*N* = 4010		*N* = 7286		OR	95% CI	*p*- value
Gender	Male	1549	39	3082	42	1.00 (ref)		
	Females	2461	61	4204	58	1.246	1.108–1.400	**0.0002**
Age (at diagnosis)	18–24 years	613	15	1168	16	1.00 (ref)		
	25–34 years	1786	45	3328	46	1.074	0.953–1.210	0.2420
	35–39 years	1611	40	2790	38	1.123	0.989–1.275	0.0734
Years since diagnosis	5–10 years	1386	35	2990	41	1.00 (ref)		
	≥11–15 years	1397	35	2496	34	1.210	1.103–1.328	**0.0001**
	≥16–20 years	1231	31	1806	25	1.489	1.348–1.645	**0.0001**
Social economic status	Low	544	14	1510	21	1.00 (ref)		
	Intermediate	1236	31	2354	32	1.406	1.245–1.587	**0.0001**
	High	2220	56	3417	47	1.763	1.575–1.974	**0.0001**
Type of cancer	Head and neck	124	3	305	4	0.933	0.711–1.224	0.6153
	Colon and rectal	82	2	151	2	1.080	0.793–1.472	0.6246
	Digestive tract, other	31	1	63	1	1.146	0.719–1.827	0.5673
	Respiratory tract	30	1	71	1	0.991	0.626–1.570	0.9696
	Melanoma	290	7	617	8	1.069	0.856–1.334	0.5576
	Breast	944	24	1553	21	1.00 (ref)		
	Female genitalia	445	11	878	12	1.042	0.864–1.257	0.6642
	Male genitalia	6	0	12	0	1.258	0.460–3.439	0.6546
	Urinary tract	46	1	105	1	1.086	0.736–1.604	0.6769
	Thyroid gland	248	6	468	6	1.213	0.976–1.508	0.0816
	Central nervous system	150	4	231	3	1.402	0.998–1.969	0.0515
	Bone or soft tissue sarcoma	172	4	291	4	1.289	1008–1.648	**0.0430**
	Germ cell tumors	692	17	1394	19	1.124	0.920–1.374	0.2532
	Lymphoid hematological malignancies	591	15	903	12	1.381	1.048–1.820	**0.0220**
	Myeloid hematological malignancies	148	4	226	3	1.294	0.877–1.908	0.1939
	Other	11	0	18	0	1.318	0.596–2.912	0.4951
Tumor stage	I	1726	43	3480	48	1.00 (ref)		
	II	1063	27	1850	25	1.026	0.916–1.149	0.6579
	III	573	14	923	13	1.174	1.022–1.348	**0.0231**
	IV	179	4	298	4	1.139	0.916–1.417	0.2412
	Missing	469	12	735	10	1.121	0.892–1.409	0.3278
Primary treatment modality	Surgery	3126	78	5863	81	1.121	0.901–1.395	0.3059
	Chemotherapy	2239	56	3632	50	1.262	1.118–1.425	**0.0002**
	Radiotherapy	1902	47	3293	45	1.030	0.935–1.135	0.5475
	Hormone therapy	484	12	786	11	0.991	0.841–1.168	0.9156
	Targeted therapy	308	8	514	7	1.013	0.862–1.191	0.8762
	Stem cell therapy	142	4	207	3	0.996	0.772–1.284	0.9738

**Table 3:** Self-reported reasons of AYA (*n* = 765) who did not want to participate in PRO research.

	*N*	%
Not interested in the research	379	49
Don’t want to think about cancer	243	32
Too busy	148	19
Questionnaire is too long/too personal/difficult	97	13
Don’t consider themself a young adult cancer patient	97	13
Have participated in research too many times	58	8
No personal incentive or benefit	32	4
Not capable to participate *	20	3
Don’t see the added value of this research	21	3
Worried about privacy aspects	16	2
Practical problems in participating **	16	2
Other ***	16	2
Unclear what causes symptoms because of comorbidities	14	2
Prefer an in person invitation	9	1
Multiple answers could be given		

* Too sick/tired/no energy, language problems, ** Log-in problems or want to participate online or just on paper, *** Felt that their situation do not contribute, didn’t appreciate study participation invitation.

## Data Availability

The data presented in this study are available on request from the corresponding author. The data are not publicly available due to privacy issues.

## Supplementary Materials

The following supporting information can be downloaded at: https://www.mdpi.com/article/10.3390/curroncol29080428/s1, Table S1: Detailed information on tumor types of the total population-based population, and divided in respondents, non-respondents and excluded.
